# Coagulation parameters in lung cancer patients: A systematic review and meta‐analysis

**DOI:** 10.1002/jcla.24550

**Published:** 2022-06-19

**Authors:** Biruk Bayleyegn, Tiruneh Adane, Solomon Getawa, Melak Aynalem, Zemen Demelash Kifle

**Affiliations:** ^1^ Department of Hematology and Immunohematology, School of Biomedical and Laboratory Sciences, College of Medicine and Health Sciences University of Gondar Gondar Ethiopia; ^2^ Department of Pharmacology, School of Pharmacy, College of Medicine and Health Science University of Gondar Gondar Ethiopia

**Keywords:** coagulation abnormalities, D‐dimer, fibrinogen, lung cancer, prothrombin

## Abstract

**Background:**

Hypercoagulability in lung cancer patients is associated with a high incidence of mortality and morbidity in the world. Therefore, this meta‐analysis aimed to explore the correlation of the basic coagulation abnormalities in lung cancer patients compared with the control.

**Method:**

PubMed, Scopus, and other sources were employed to identify eligible studies. The outcome variable was expressed using mean ± standard deviation (SD). Heterogeneity among studies and publication bias were evaluated. The quality of included studies was also assessed based on Newcastle–Ottawa Scale checklist.

**Result:**

Finally, through a total of eight studies, prolonged prothrombin time (PT; standard mean difference [SMD]: 1.29; 95% CI: 0.47–2.11), plasma D‐dimer value (SMD 3.10; 95% CI 2.08–4.12), fibrinogen (SMD 2.18; 95% CI:1.30–3.06), and platelet (PLT) count (SMD 1.00; 95% CI 0.84–1.16) were significantly higher in lung cancer patients when compared with the control group. The single‐arm meta‐analysis also showed that compared with control, lung cancer patients had high pooled PT 13.7 (95% CI:12.2–15.58) versus 11.79 (95% CI = 10.56–13.02), high D‐dimer 275.99 (95% CI:172.9–11735.9) versus 0.2 (95% CI:0.20–0.37), high plasma fibrinogen 5.50 (95% CI:4.21–6.79) versus 2.5 (95% CI:2.04–2.91), and high PLT count 342.3 (95% CI:236.1–448.5) versus 206.6 (95% CI:176.4–236.7).

**Conclusion:**

In conclusion, almost all the coagulation abnormalities were closely associated with lung cancer, and hence coagulation indexes provide an urgent clue for early diagnosis and timely management.

## Graphical abstract

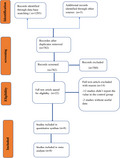

## 1 INTRODUCTION

Lung cancer is the leading cause of cancer‐related death worldwide and is the second most common cancer diagnosis in men and women (after prostate and breast cancer, respectively). By the year 2018, about 2 million new cases of lung cancer were diagnosed and 1.8 million lung cancer‐related deaths were reported worldwide. It has the highest incidence in developing nations where cigarette smoking is most prevalent,^1^ and there is a focus on prevention, early detection, and development of new treatment options that impact the epidemiological patterns of lung cancer globally.^2^

Lung cancer is caused by numerous factors such as radon gas, asbestos, air pollution exposures, chronic infections, as well as genetic susceptibility. However, 90% of lung cancers are caused by smoking and the use of tobacco products.^3^ Based on cell origin, which grows and spreads differently, lung cancer is classified into two main histological groups: small cell lung carcinoma (15% of all lung cancers) and non‐small cell lung carcinomas (NSCLC, 85% of all lung cancers).^4^

Ample of evidence has reported that lung cancer is associated with hypercoagulability by involving activation of coagulation and the fibrinolytic system at clinical or subclinical levels.^5^ Hypercoagulability is increased coagulation, decreased anticoagulation function, and decreased tissue fibrinolytic activity^6^ caused by a variety of factors, that tmour cells can directly activate blood clotting, inducing active production of procoagulant, suppressing anticoagulant, increased platelets (PLT), monocytes, and macrophages.^7^

Fibrinogen is the most abundant plasma coagulation factor synthesized mainly by hepatic cells. High level of plasma fibrinogen level was detected in lung cancer patients.^8^ Even though, the prognostic role of plasma fibrinogen in lung cancer remains controversial,^9^ many studies have shown that a high level of fibrinogen (>400 mg/dl) is one of the most important indicators of poor progression and worse overall survival in patients with lung cancer and serves as a prognostic biomarker.^8^, ^10^, ^11^

Furthermore, the other key indicator associated with mortality in lung cancer patients is increased D‐dimer, the degradation product of fibrin, which signals hyperfibrinolysis in response to clot activation and fibrin formation.^12^ Several studies have revealed that the elevated D‐dimer levels were significantly associated with shorter survival time and early recurrence in lung cancer patients than those with normal levels.^13^, ^14^, ^15^ Moreover, a meta‐analysis conducted by *Xi Zhang* and *Yuge Ran* showed that thrombocytosis was recognized as predictor of high‐risk progression and poor prognosis in lung cancer patients.^16^

Notably, coagulation function indexes include prolonged prothrombin time (PT), activated partial thromboplastin time (APTT), international normalized ratio (INR), increased D‐dimer, and fibrinogen, as well as PLT count, are independent prognostic markers of lung cancer. However, the results remain inconclusive among studies. Therefore, the aim of this systematic review and meta‐analysis was to delineate the correlation of basic coagulation abnormalities with lung cancer patients as compared to normal controls. Hence, this aggregate of data will help health‐care providers to confine the lung cancer complication by early detection and proper management nationwide.

## 2 METHODS AND MATERIALS

### 2.1 Eligibility criteria

Regarding the language restriction, all studies included in this study were published in English. Whereas articles without study outcomes, lacking control groups, and studies did not report in the form of mean ± SD or median‐interquartile range (IQR), as well as comparative studies between before and after treatment, were excluded. Additionally, studies not presenting original data such as systematic reviews, meta‐analyses, case–control, and poster presentation were excluded from the study.

### 2.2 Data source and searching strategy

This systematic review was conducted following the PRISM2020 guideline^17^ (Appendix S1 PRISMA checklist). Previously published articles were systematically and vigorously searched using major electronic databases including PubMed, Scopus, and Google Scholar in the period from inception up to October 2021. Besides, the reference lists of selected studies were also checked for identifying additional relevant studies. The search terms were used separately and in combination using Boolean operators like “OR” or “AND”. The search terms were (“coagulation” OR “coagulation abnormality” OR “coagulation profile” OR “coagulation parameters” OR “blood coagulation” OR “hemostatic” OR “haemostatic” OR “Prothrombin time” OR “activated partial thrombin time” OR “D‐dimer” OR “fibrinogen” OR “platelet count”) AND (“Lung cancer”; Appendix S2 Search strategy).

### 2.3 Study selection and data extraction

Based on the abovementioned eligibility criteria, two independent investigators separately retrieved the publication after the screening of the title, abstract, and full text, and evaluated the eligible studies. Any discrepancies from the investigators were resolved by consulting the third investigator. Extracted data elements from each included study were the name of the first author, country, publication year, study design, total sample size, and sample size from the two wings, the value of PT, APTT, INR, D‐dimer, fibrinogen, and PLT count. Mean ± SD was applied to present the continuous variables. Whereas any variables represented by median and IQR were converted to the form of mean ± SD accordingly.^18^

### 2.4 Assessment of methodological quality

The quality of included studies was assessed based on the NOS checklist for case–control studies. The NOS checklist for case–control studies were categorized into three main components including selection, comparability, and exposure and each of them contains 4, 1, and 3 questions, respectively. One star* and no star* were awarded for each equation, which were assigned 1 and 0, respectively. Then, a study with a score ≥7 was considered a high‐quality study^19^ (Appendix S3 Quality Assessment).

### 2.5 Data synthesis and analysis

All selected studies were managed using EndNote version 9.2 software and duplicates of studies were removed. All the statistical analyses were performed with the STATA software version 11.0. Standard mean difference (SMD) with a 95% confidence interval (CI) was analyzed as a prognostic indicator of each prolonged coagulation values in the lung cancer patients. The combined SMD >0 and its 95% CI did not overlap 0 indicating that a higher level of PT, APTT, INR, plasma fibrinogen, and D‐dimer could be a poorer prognostic factor in lung cancer patients, whereas the combined SMD <0 and 95% CI contain 0 this means that the *p*‐value >0.05 indicated better prognosis.^20^

Statistical heterogeneity of included studies was determined with the I^2^ index.^21^ *I* ^2^ was employed to evaluate statistical heterogeneity with *I* ^2^ values of <25%, 25%–75%, and >75%, respectively, indicating low, moderate, and high heterogeneity. *I* ^2^ test is an indicator for the choice of the proper effect model whether the fixed effect model or random effect model was applied. *I* ^2^ < 50% or *p*‐value <0.10 were considered no significant heterogeneity between studies, and thus the fixed‐effects model was applied. Otherwise, the random‐effects model was applied. Furthermore, sensitivity analysis was performed to validate the stability of the pooled results by omitting the studies one by one. Meta‐regression was also conducted to explore the effect of continuous covariates on differences in the coagulation parameters between lung cancer patients and control groups.

### 2.6 Risk of bias

The potential for publication bias was assessed by performing a funnel plot test. Publication bias was determined by the shape of the funnel plot, where a symmetrical shape represented relatively low publication bias, while an asymmetrical shape indicated relatively high publication bias. Meanwhile, Eggers's regression test was applied to make a quantitative analysis of publication bias, and a *p*‐value <0.05 was used to indicate a statistically significant publication bias between included studies.

## 3 RESULTS

### 3.1 Study selection and characteristics

**FIGURE 1:**
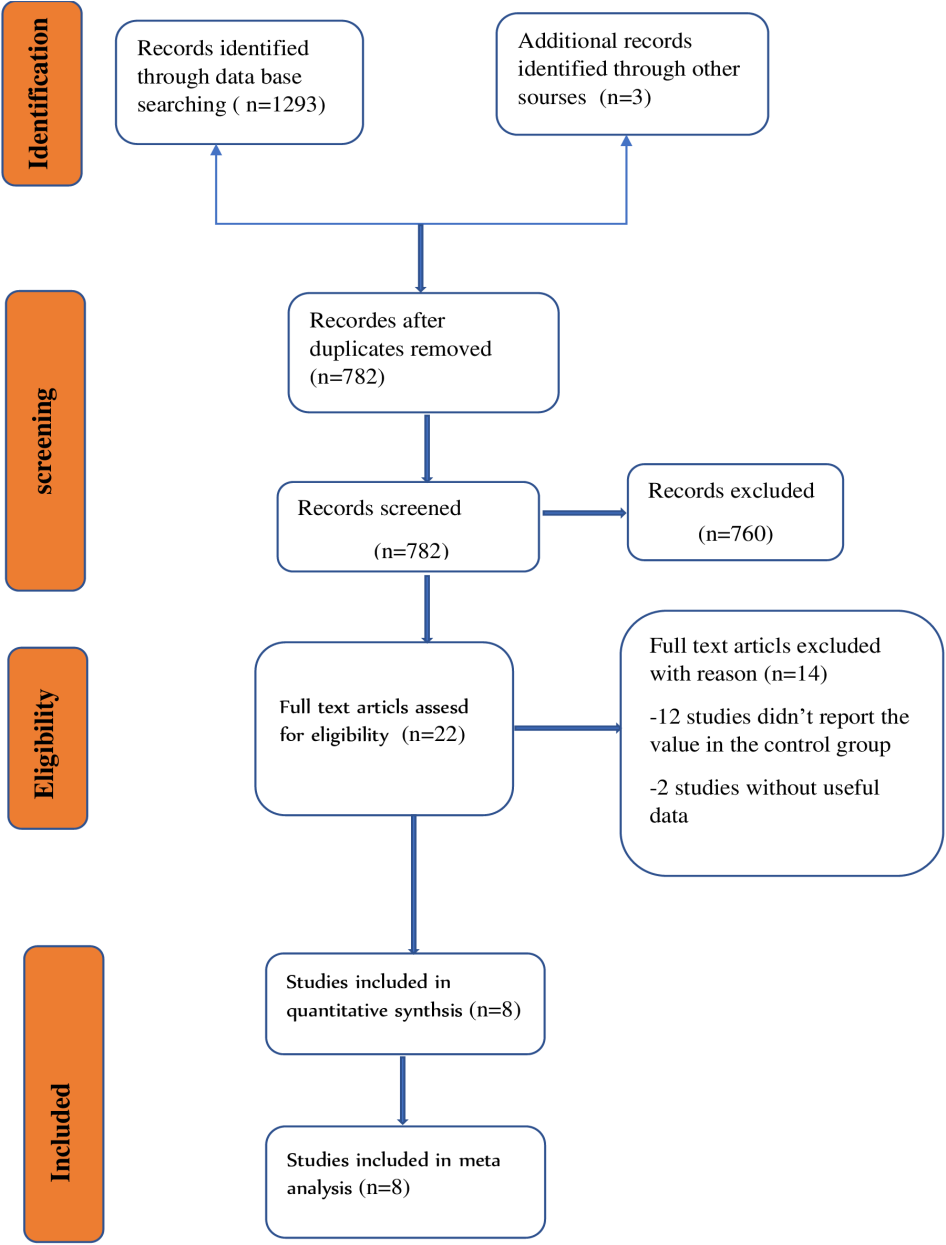
Flowchart of search stratagem and study selection

A total of 1296 articles were initially identified through the PubMed database and other sources. After eliminating duplicate articles as well as detailed reading of the title and abstract of each article, 22 relevant articles were screened out for full‐text assessment based on eligibility criteria. Finally, for the pooled analysis, eight studies were included (Figure 1).

The detailed characterstics of included studies were summarized in Table 1. Included studies originated from four countries and all included studies were case–control type of study design between lung cancer and normal control group. In this meta‐analysis, there were a total of 1886 study participants and from those 1528 and 358 were the lung cancer patients and control groups, respectively. All studies did not report all the required outcomes in the meantime. Besides, PT value were reported by all included studies among case and control groups. However, PLT count and INR value were not reported by two of the included studies whereas only one study did not report the plasma D‐dimer level between the case and control group. Regarding plasma fibrinogen, only five included studies reported the value of fibrinogen among lung cancer patients and control participants.

**TABLE 1:** Basic charcterstics of included studies for the detemination of coagulation abnormality in lung cancer

Author	Area	Study design	Sample size	PLT	PT	APTT	INR	D‐D	Fibrinogen
Ujjan et al., 2009^17^	Pakistan	Case–control	Case 40	394 ± 170	14.7 ± 0.5	41.5 ± 6.2			
			Control 30	216 ± 73	12.6 ± 0.4,	25.8 ± 3.7			
Komurcuoglu et al., 2011(^40^)	Turkey	Case–control	Case 100	377.3 ± 134	13.63 ± 1.4	31.81 ± 3.96	1.15 ± 0.17	1076.66 ± 10.41 ng/dl	
			Control 25	258 ± 112	11.6 ± 1.2	29.2 ± 3.1	0.98 ± 0.11	248.6 ± 116	
Inal et al., 2015 (^41^)	Turkey	Case–control	Case 72		15.6 ± 2.1		1.3 ± 0.4	1321 ± 734.5 ng/dl	
			Control 40		12.8 ± 1.3		0,8 ± 0.2	379.5 ± 174.5 ng/dl	
Yanhua et al., 2014 (^42^)	China	Case–control	Case 604	336.8 ± 136.3	14.7 ± 4.3	35.1 ± 8.6	1.3 ± 0.3	2.2 ± 2.0 mg/L	5.2 ± 2.0
			Control 50	200 ± 32	9.9 ± 0.5	32 ± 0.9	0.9 ± 0.03	0.1 ± 0.02	2.6 ± 0.5
Yongjun et al., 2017 (^43^)	China	Case–control	Case 539	335.3 ± 137.8	19.9 ± 4.5	34.8 ± 8.5	1.3 ± 0.3	2.3 ± 2.1 mg/L	7.2 ± 2.0
			Control 80	196.3 ± 34.3	9.8 ± 0.6	31.5 ± 0.6	0.9 ± 0.03	0.2 ± 0.05	2.48 ± 0.45
Tas et al., 2021^23^	Turkey	Case–control	Case 110	342.3 ± 157.3	14.4 ± 2.4	29.5 ± 6.1	1.4 ± 0.5	1660.5 ± 1469.5 IU/ml	6.64 ± 3.94
			Control 50	204.8 ± 22.8	13.8 ± 1.6	38.6 ± 5.9	1.01 ± 0.1	48.4 ± 29.4	2.80 ± 0.74
van Wersch et al., 1991(^44^)	Netherlands	Case–control	Case 48	313 ± 98.3	14.7 ± 2.3	29.4 ± 3.1		2677 ± 4622 ng/ml	4.9 ± 1.4
			Control 50	265 ± 68	13 ± 1	28 ± 2		238 ± 100	2.9 ± 0.6
Wang et al., 2016 (^45^)	China	Case–control	Case 15		9.69 ± 1.42	30.14 ± 5.79	0.76 ± 0.10	0.84 ± 0.17 mg/L	5.43 ± 0.89
			Contole 33		13.1 ± 1.96	39.34 ± 7.63	1.35 ± 0.22	0.30 ± 0.06	2.14 ± 0.36

### 3.2 Meta‐analysis of coagulation indicators between lung cancer and control

**FIGURE 2:**
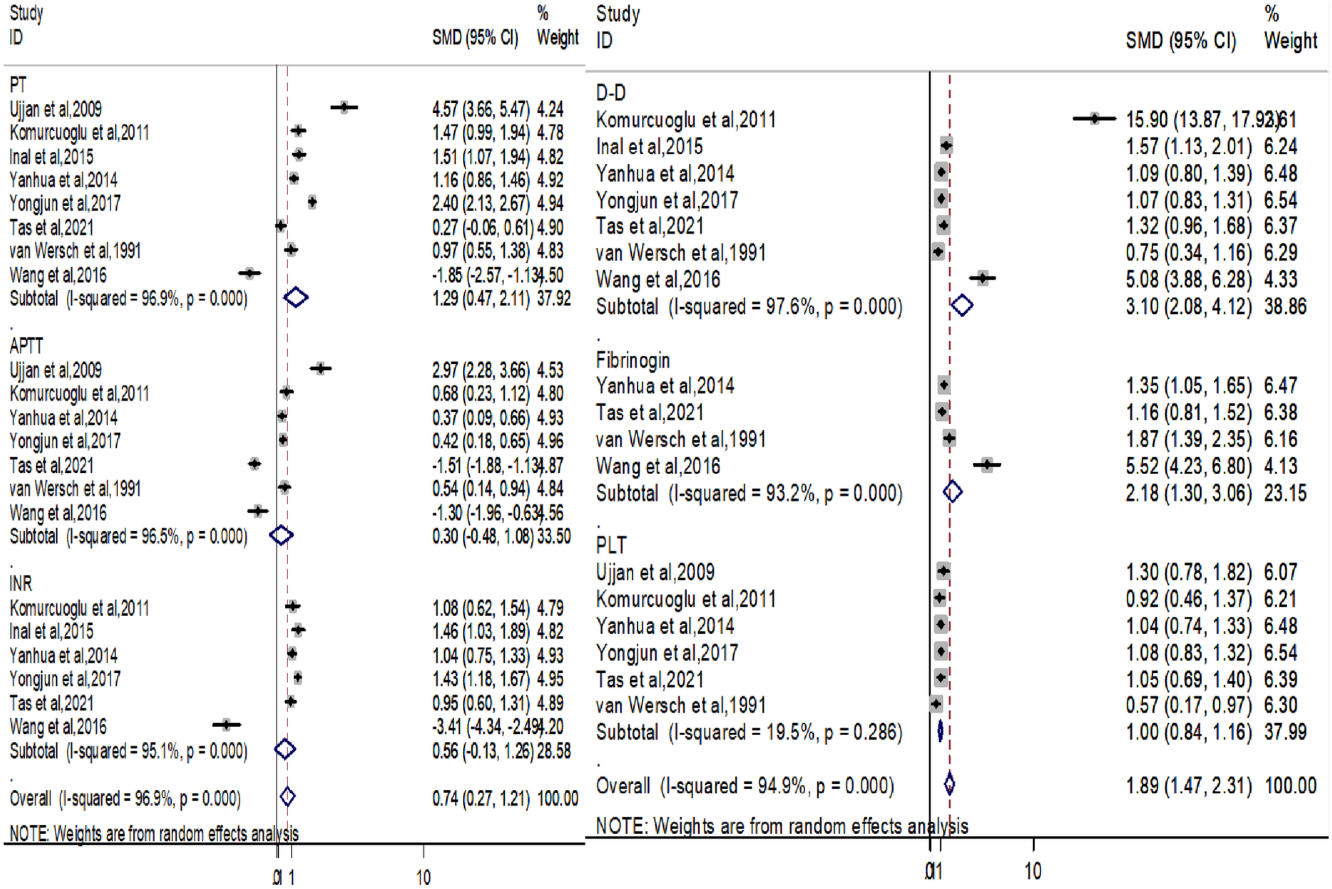
Forest plot of the value of PT (prothrombin), APTT (activated partial thrombin time), INR, D‐D (D‐dimer), and fibrinogen, PLT (platelet) in lung cancer patients compared with control. SMD, Standard mean difference

Among enrolled studies, six coagulation indicators including PT, APTT, INR, D‐dimer, fibrinogen, and PLT were analyzed and compared between lung cancer and control groups (Figure 2).

**FIGURE 3:**
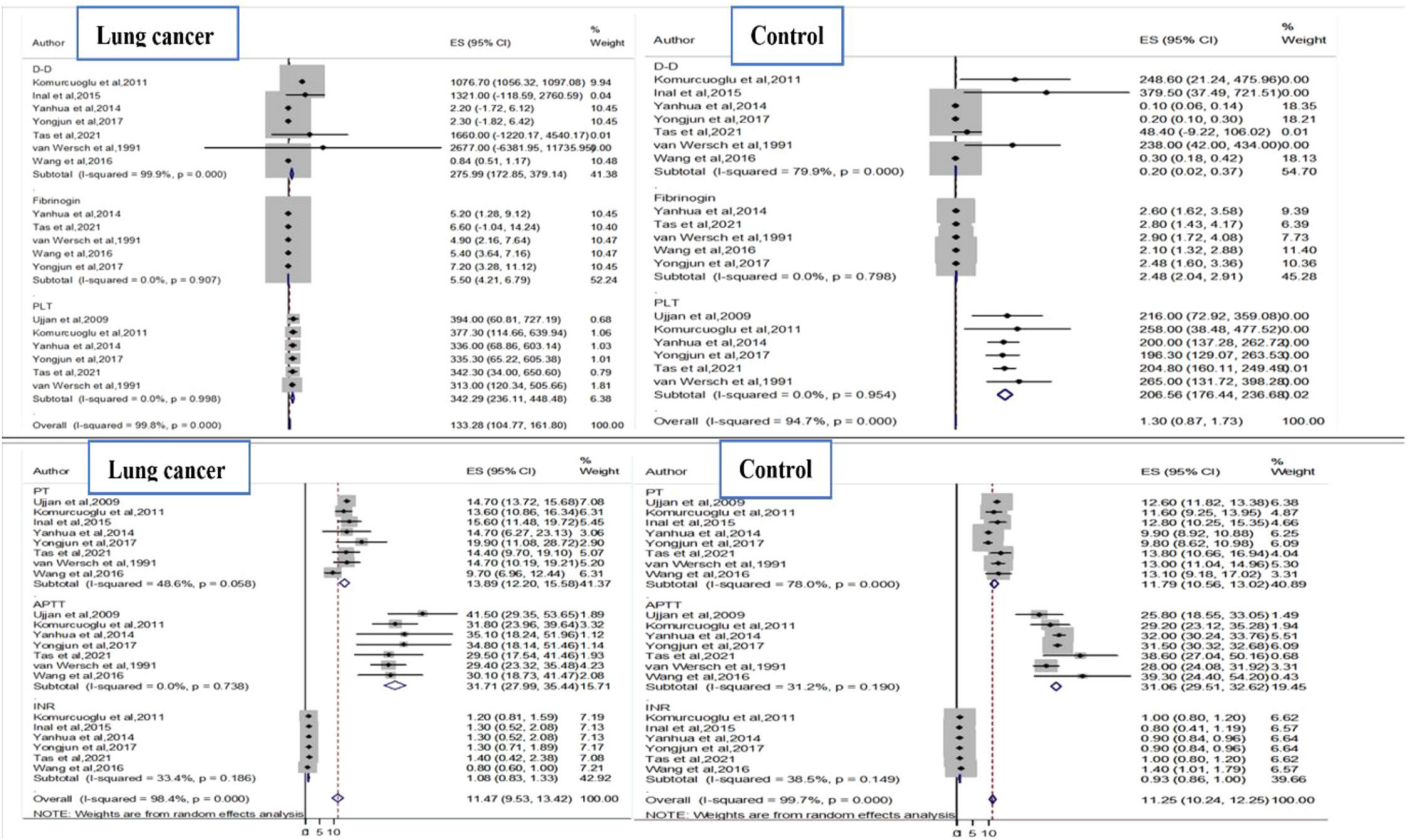
Forest plot for the pooled value of PT (prothrombin), APTT (activated partial thrombin time), INR, D‐D (D‐dimer), fibrinogen, PLT (platelet) in lung cancer patient and control. ES, effect size

Moreover, the single‐arm meta‐analysis showed the pooled value of selected coagulation indicators among lung cancer and normal control (Figure 3).

#### 3.2.1 PT between lung cancer and control

For the determination of PT, all enrolled studies were eligible. According to the *I* ^2^ value (96.9%), the random effect model was adopted for the statistical analysis of PT. The result showed that prolonged PT was significantly higher in lung cancer patients than in control groups (SMD: 1.29; 95% CI: 0.47–2.11; *p* = 0.000; Figure 2). According to single‐arm meta‐analysis, the pooled PT was 13.7 (95% CI:12.2–15.58) among lung cancer patients but 11.79 (95% CI = 10.56–13.02) was among the control group (Figure 3).

#### 3.2.2 APTT between lung cancer and control

A total of 1456 lung cancer patients and 318 controls were involved in the determination of APTT. The statistics showed that prolonged APTT was higher among lung cancer patients than its counterparts (SMD 0.30; 95% CI −0.48–1.08; *p* = 0.000; Figure 2) without significant difference.

#### 3.2.3 INR between lung cancer and control

Six studies with 1440 lung cancer patients and 278 control were enrolled for meta‐analysis. Accordingly, using random‐effect model with obviously high heterogeneity (*I* ^2^ = 95.1%), INR values were higher among lung cancer patients than the control group (SMD 0.56; 95% CI: −0.13‐1.26; *p* = 0.000; Figure 2) but the difference was not significant.

#### 3.2.4 D‐dimer level between lung cancer and control

Plasma D‐dimer was assessed using 7 studies with a total of 1488 lung cancer patients and 328 control groups. The random effect model with high heterogeneity (*I* ^2^ = 97.6%) demonstrated that the plasma D‐dimer value was significantly higher in lung cancer patients (SMD 3.10; 95% CI 2.08–4.12; *p* = 0.000) when compared with the control group (Figure 2). The single‐arm meta‐analysis of 7 studies revealed that the pooled D‐dimer was 275.99 (95% CI:172.9–11735.9) as compared with the control group 0.2 (95% CI:0.20–0.37; Figure 3).

#### 3.2.5 Fibrinogen between lung cancer and control

To compare the plasma fibrinogen among lung cancer and control groups, five studies were involved with a total of 1316 lung cancer patients and 263 control groups. The meta‐analysis showed that plasma fibrinogen was significantly higher in lung cancer patients (SMD 2.18; 95% CI:1.30–3.06; *p* = 0.000) as compared with the control with high heterogeneity (*I* ^2^ = 93.2%) in the random effect model (Figure 2). The pooled plasma fibrinogen among lung cancer patients was 5.50 (95% CI:4.21–6.79) based on the single‐arm meta‐analysis and 2.5 (95% CI:2.04–2.91) was recorded from the control group (Figure 3).

#### 3.2.6 PLT count between lung cancer and control

In this meta‐analysis, PLT count was assessed using six studies with 1441 lung cancer patients and 285 control groups. According to the random effect model with minimal heterogeneity (*I* ^2^ = 19.5%), the PLT count was significantly higher in lung cancer patients than the control group (SMD 1.00; 95% CI 0.84–1.16; *p* = 0.286; Figure 2). Results from single‐arm meta‐analysis showed that the pooled PLT count in the lung cancer patient was 342.3 (95% CI:236.1–448.5) whereas 206.6 (95% CI:176.4–236.7) in the normal control group (Figure 3).

### 3.3 Publication bias

The potential for publication bias was assessed by a visual inspection of the funnel test. The meta‐analysis of Begg's funnel plot demonstrated no evidence of publication bias was present for PLT count, PT, and APTT with *p*‐value of 0.713, 0.744, and 0.886, respectively. However, there was significant publication bias among enrolled studies to determine INR (*p*‐value = 0.055), D‐dimer (*p*‐value = 0.009), and fibrinogen (*p*‐value = 0.041; Figure S1).

For further quantitative analysis, we conducted Egger's regression test and confirmed that there was no significant statistical evidence of publication bias among enrolled studies for PLT count, PT, and APTT analysis (Table S1 Eggers test).

### 3.4 Sensitivity analysis

Sensitivity analysis was conducted to evaluate the effect of each study on the overall result reliability. Thus, sensitivity analysis suggested that these meta‐analyses (PLT count, D‐dimer, APTT, PT, INR, and fibrinogen) were steady with the exclusion of any of the studies (Table S2 Sensitivity analysis).

### 3.5 Meta‐regression

After conducting meta‐regression by considering the publication year, the result demonstrated that all coagulation indicators among lung cancer patients were not associated with the year of publication (Table 2).

**TABLE 2:** Meta regression

Variable	PLT		PT		APTT		INR		D‐dimer		Fibrinogen	
	Coefficient	*p*‐value	Coefficient	*p*‐value	Coefficient	*p*‐value	Coefficient	*p*‐value	Coefficient	*p*‐value	Coefficient	*p*‐value
publication year	0.016	0.12	−0.031	0.71	−0.061	0.37	−0.033	0.91	0.016	0.95	0.017	0.85

## 4 DISCUSSION

Blood coagulation function indexes are closely important for the clinical decision of palliative chemotherapy.^22^ However, there is no well‐standardized information about all‐coagulation parameters in lung cancer patients. Hence, the current meta‐analysis provides a comprehensive clue about coagulation indicators as a prognostic marker for lung cancer patients.

In this meta‐analysis, six coagulation indicators (PT, APTT, INR, D‐dimer, fibrinogen, and PLT count) were evaluated to assess the difference in coagulation abnormality among lung cancer patients and the normal control group. The result showed that prolonged PT, increased PLT count, high level of plasma D‐dimer, as well as high plasma fibrinogen, were significantly higher in lung cancer patients than in control groups. However, prolonged APTT and INR were higher in lung cancer patients than the control group without significant differences. Prolongation of PT was significantly higher and associated with poor prognosis among lung cancer patients as compared with normal control in this meta‐analysis. This prolonged PT signifies a deficiency of coagulation factors or the presence of specific factor inhibitors involved in the coagulation cascade.^23^

Hypercoagulability was the most common event in lung cancer patients.^5^ The mechanism of the high incidence of hypercoagulability in cancer patients is due to the involvement of several coagulation factors and signaling pathways in the process. Cancer cells can express procoagulant factors, including tissue factor, cancer procoagulant, and heparanase, which are important for activation of the coagulation cascade. Cancer cells can also secrete soluble mediators such as tumor necrosis factor‐a, interleukin‐1b, or make direct contact with the host vascular and blood cells to stimulate the expression of procoagulant factors in these cells.^24^

The relationship and underlying mechanisms between increased PLT count and lung cancer were not well described yet. Despite this fact, PLT plays a significant role in cancer cell growth, progression, and metastasis. First, PLT releases various cytokines, including vascular endothelial growth factor and PLT ‐derived growth factor, from a‐granules, dense granules, and lysosomes, which affect tumor cell proliferation and angiogenic activity. Moreover, PLT promote the formation of the capillary‐like structure by interacting with vascular endothelial cells, via tumor cell surface integrins mediating cell–cell adhesion. But blocking of integrins GPIIb/IIIa and inhibition of endothelial ab3 integrins significantly decreased angiogenesis and subsequently reduced tumor cell growth.^25^ Secondly, PLT enhance tumor metastasis by protecting the tumor cells from the host's immune system. This is by the mechanism that PLT expressing different immunoregulatory proteins, including glucocorticoid‐induced TNFR‐related protein, may protect cancer cells. The inhibition of PLT activation significantly decreases the metastatic potential of tumor cells.^26^

The finding from this meta‐analysis was consistent with other meta‐analyses published previously that showed elevated PLT counts were significantly associated with poor prognosis among lung cancer patients.^27^ Recent evidence argues that PLT count is considered a hallmark of cancer and is correlated with prognosis in different malignancies such as renal, gastric, colorectal, hepatocellular, and lung cancer.^28^ Another meta‐analysis with 20 enrolled studies indicates that the relationship between pre‐treatment thrombocytosis and lung cancer is a generic future across all lung cancer types and that thrombocytosis is useful diagnostical indicator of all major subtypes of lung cancer.^29^

Many studies showed that the elevation of plasm fibrinogen was predictive of thromboembolic disease at the early diagnosis of lung cancer before any therapy.^30^, ^31^ Then, plasma fibrinogen between lung cancer patients and the control group was the other coagulation abnormalities indicators analyzed in this meta‐analysis using five studies with a total of 1316 lung cancer patients and 263 control groups. This result showed that plasma fibrinogen was significantly higher in lung cancer patients as compared with the control. The finding is supported by other meta‐analyses published previously by including a total of 17 studies with 6460 lung cancer patients and the study showed that high plasma fibrinogen levels predicted a worse prognosis in lung cancer patients.^30^ Additionally, other meta‐analyses by 16 studies demonstrated that elevated plasma fibrinogen could significantly predict the poor prognosis of lung cancer.^9^

Even though the clear pathophysiological mechanism of the prognostic significance of higher plasma fibrinogen levels in lung cancer patients was not well understood, recent studies have demonstrated that fibrinogen could play a key role in tumor progression.^32^ First fibrinogen may serve as a reservoir, controlling growth factor bioavailability and accessibility, and influencing cancer cell proliferation, inhibition of apoptosis, angiogenesis, and metastases. Furthermore, fibrinogen has been implicated in the formation of tumor stroma, which provides gas exchange and nutrients for rapidly proliferating tumor cells.^32^, ^33^ Second, tumor cells were capable of endogenous production of fibrinogen, and that fibrinogen could bind to some growth factors, such as vascular endothelial growth factor and fibroblast growth factor, and facilitate these growth factors binding to their receptors on the tumor cell surface, which plays a crucial role in contributing to tumor proliferation and angiogenesis.^34^, ^35^ Fibrinogen‐mediated cellular bridging may provide traction for cancer cell adhesion, shape changes, motility, and invasive potential.^33^ Third, fibrinogen deposition with the help of thrombin and PLT could promote thrombosis by strengthening the interaction of cancer cells, assisting tumor cells in escaping from being killed by natural killers.^36^ This all concluded that as fibrinogen could promote tumor migration, metastasis, and angiogenesis, it could become a prognostic factor for lung cancer.

The tumor microenvironment constituted by immune cells and stromal cells with other “niche” cells leads to cancer patients' tumor‐associated coagulation abnormalities, which increases plasma D‐dimer concentration, one of the fibrin degradation products. Furthermore, D‐dimer is associated with lymph node metastases in NSCLC.^37^ This meta‐analysis points out that the plasma D‐dimer value was significantly higher in lung cancer patients (SMD 3.10; 95% CI 2.08–4.12; *p* = 0.000) when compared with the control group using seven studies. The single‐arm meta‐analysis on seven studies with a total of 1488 lung cancer patients and 328 control groups revealed that the pooled D‐dimer was 275.99 (95% CI:172.9–11735.9) as compared with the control group 0.2 (95% CI:0.20–0.37). The finding is similar to another meta‐analysis conducted by Ma et al. which showed that high plasma D‐dimer level is an independent factor of poor prognosis in patients with lung cancer.^38^ Additionally, the meta‐analysis conducted by Tumor et al. by 11 enrolled studies suggested that a higher plasma D‐dimer level was associated with a poor prognosis of lung cancer.^39^ The main limitation of this meta‐analysis was almost all studies included in this meta‐analysis were published in English which lacks the conclusiveness of studies written in other languages.

## 5 CONCLUSION

This meta‐analysis pointed out that prolonged PT, increased PLT count, and high levels of plasma D‐dimer and plasma fibrinogen were significantly higher in lung cancer patients than in control groups, which provides the hallmark indicators of lung cancer disease. Therefore, in the future, physician pays attention to the early screening of coagulation function indexes for lung cancer patients to reduce further complications.

## AUTHOR CONTRIBUTIONS

BB; Conceptualized, designed the study, and write‐up all the manuscript. TA and SG; accomplished the statistical analysis as well as critically revised the manuscript. MA: provided supervision and critically revised the manuscript. All authors approved the final version of the manuscript and agreed accountability for all aspects of the manuscript.

## CONFLICT OF INTEREST

All the authors declared that no conflict of interest for this work.

## Supporting information


Figure S1

Table S1

Table S2

Appendix S1

Appendix S2

Appendix S3

## Data Availability

All the required data were fully available within the manuscript without any restriction.

## References

[1] Thandra KC , Barsouk A , Saginala K , Aluru JS , Barsouk A . Epidemiology of lung cancer. Contemp Oncol. 2021;25(1):45. 10.5114/wo.2021.103829 PMC8063897 33911981

[2] Jakobsen E , Olsen KE , Bliddal M , Hornbak M , Persson GS , Green A . Forecasting lung cancer incidence, mortality, and prevalence to year 2030. BMC Cancer. 2021;21(1):985. 34479490 10.1186/s12885-021-08696-6 PMC8414713

[3] Alaoui LHHO , Yi Y , Buchanana P . Lung cancer: biology and treatment options. Biochim Biophys Acta. 2015;1856(2):189‐210. 26297204 10.1016/j.bbcan.2015.08.002 PMC4663145

[4] Inamura K . Lung cancer: understanding its molecular pathology and the 2015 WHO classification. Front Oncol. 2017;7:193. 28894699 10.3389/fonc.2017.00193 PMC5581350

[5] Hammouda ASS , Hamida FYM , Amir CZ , Bouguerra AS , Hariti G . Activation of coagulation in patients with lung cancer. Ann Biol Clin. 2019;77(3):272‐280. 10.1684/abc.2019.1445 31219420

[6] Ma Y , Li G , Li X , et al. Clinical characteristics and prognostic analysis of lung cancer patients with hypercoagulability: a single‐center, retrospective, real‐world study. J Cancer. 2021;12(10):2968‐2974. 33854597 10.7150/jca.46600 PMC8040890

[7] Kadlec B , Skrickova J , Merta Z , Dusek L , Jarkovsky J . The incidence and predictors of thromboembolic events in patients with lung cancer. Sci World J. 2014;2014:1‐9. 10.1155/2014/125706 PMC3918375 24574864

[8] Liu X , Shi B . Progress in research on the role of fibrinogen in lung cancer. Open Life Sci. 2020;15(1):326‐330. 33817221 10.1515/biol-2020-0035 PMC7874584

[9] Zhong H , Qian Y , Fang S , Wang Y , Tang Y , Gu W . Prognostic value of plasma fibrinogen in lung cancer patients: a meta‐analysis. J Cancer. 2018;9(21):3904‐3911. 30410594 10.7150/jca.26360 PMC6218779

[10] Bian N‐N , Shi X‐Y , Qi H‐Y , et al. The relationship of plasma fibrinogen with clinicopathological stages and tumor markers in patients with non‐small cell lung cancer. Medicine. 2019;98(32):e16764. 31393394 10.1097/MD.0000000000016764 PMC6708950

[11] Fan S , Guan Y , Zhao G , An G . Association between plasma fibrinogen and survival in patients with small‐cell lung carcinoma. Thorac Cancer. 2018;9(1):146‐151. 29131503 10.1111/1759-7714.12556 PMC5754299

[12] Jiang X , Mei X , Wu H , Chen X . D‐dimer level is related to the prognosis of patients with small cell lung cancer. Ann Transl Med. 2017;5(20):394. 29152494 10.21037/atm.2017.07.35 PMC5673792

[13] Shiina Y , Nakajima T , Yamamoto T , et al. The D‐dimer level predicts the postoperative prognosis in patients with non‐small cell lung cancer. Plos One. 2019;14(12):e0222050. 31877562 10.1371/journal.pone.0222050 PMC6932866

[14] Chen Y , Yu H , Wu C , et al. Prognostic value of plasma D‐dimer levels in patients with small‐cell lung cancer. Biomed Pharmacother. 2016;81:210‐217. 27261596 10.1016/j.biopha.2016.02.030

[15] Wang Y , Wang Z . Predictive value of plasma D‐dimer levels in patients with advanced non‐small‐cell lung cancer. Onco Targets Ther. 2015;8:805. 25926741 10.2147/OTT.S78154 PMC4403698

[16] Zhang X , Ran Y . Prognostic role of elevated platelet count in patients with lung cancer: a systematic review and meta‐analysis. Int J Clin Exp Med. 2015;8(4):5379‐5387. 26131114 PMC4483921

[17] Ujjan ID , Khokhar NA , Shaikh MA , Shaikh IA , Memon RA , Maheshwari N . Evaluation of coagulation abnormalities in lung cancer patients. JLUMHS. 2009;8(02):118.

[18] Wan X , Wang W , Liu J , Tong T . Estimating the sample mean and standard deviation from the sample size, median, range and/or interquartile range. BMC Med Res Methodol. 2014;14(1):1‐13. 25524443 10.1186/1471-2288-14-135 PMC4383202

[19] Stang A . Critical evaluation of the Newcastle‐Ottawa scale for the assessment of the quality of nonrandomized studies in meta‐analyses. Eur J Epidemiol. 2010;25(9):603‐605. 20652370 10.1007/s10654-010-9491-z

[20] Tan SH , Tan SB . The correct interpretation of confidence intervals. Proc Singapore Healthc. 2010;19(3):276‐278.

[21] von Hippel PT . The heterogeneity statistic I 2 can be biased in small meta‐analyses. BMC Med Res Methodol. 2015;15(1):1‐8. 25880989 10.1186/s12874-015-0024-z PMC4410499

[22] Abbas M , Kassim SA , Wang ZC , Shi M , Hu Y , Zhu HL . Clinical evaluation of plasma coagulation parameters in patients with advanced‐stage non‐small cell lung cancer treated with palliative chemotherapy in China. Int J Clin Pract. 2020;74(12):e13619. 32726491 10.1111/ijcp.13619

[23] Tas F , Kilic L , Serilmez M , Keskin S , Sen F , Duranyildiz D . Clinical and prognostic significance of coagulation assays in lung cancer. Respir Med. 2013;107(3):451‐457. 23200643 10.1016/j.rmed.2012.11.007

[24] Zhang M , Wu S , Hu C . Do lung cancer patients require routine anticoagulation treatment? A meta‐analysis. J Int Med Res 2020;48(1):0300060519896919. 31948304 10.1177/0300060519896919 PMC7113707

[25] Ji Y , Sheng L , Du X , Qiu G , Su D . Elevated platelet count is a strong predictor of poor prognosis in stage I non‐small cell lung cancer patients. Platelets. 2015;26(2):138‐142. 24679181 10.3109/09537104.2014.888547

[26] Dangfan Y , Bingjiang L , Lizhen Z , Kaiqi D . Platelet count predicts prognosis in operable non‐small cell lung cancer. Exp Ther Med. 2013;5(5):1351‐1354. 23737877 10.3892/etm.2013.1003 PMC3671769

[27] Yuan Y , Zhong H , Ye L , et al. Prognostic value of pretreatment platelet counts in lung cancer: a systematic review and meta‐analysis. BMC Pulm Med. 2020;20(1):1‐11. 32312252 10.1186/s12890-020-1139-5 PMC7171794

[28] Wang Y‐H , Kang J‐K , Zhi Y‐F , et al. The pretreatment thrombocytosis as one of prognostic factors for gastric cancer: a systematic review and meta‐analysis. Int J Surg. 2018;53:304‐311. 29654963 10.1016/j.ijsu.2018.03.084

[29] Barlow M , Hamilton W , Ukoumunne OC , Bailey SE . The association between thrombocytosis and subtype of lung cancer: a systematic review and meta‐analysis. Transl Cancer Res. 2021;10(3):1249‐1260. 35116452 10.21037/tcr-20-3287 PMC8798371

[30] Zhang Y , Cao J , Deng Y , et al. Pretreatment plasma fibrinogen level as a prognostic biomarker for patients with lung cancer. Clinics. 2020;75:e993. 32130355 10.6061/clinics/2020/e993 PMC7026942

[31] Ohara S , Suda K , Tomizawa K , et al. Prognostic value of plasma fibrinogen and D‐dimer levels in patients with surgically resected non‐small cell lung cancer. Surg Today. 2020;50(11):1427‐1433. 32409869 10.1007/s00595-020-02019-1

[32] Chen P , Wang C , Cheng B , et al. Plasma fibrinogen and serum albumin levels (FA score) act as a promising prognostic indicator in non‐small cell lung cancer. Onco Targets Ther. 2017;10:3107‐3118. 28790844 10.2147/OTT.S138854 PMC5488757

[33] Simpson‐Haidaris P , Rybarczyk B . Tumors and fibrinogen: the role of fibrinogen as an extracellular matrix protein. Ann NY Acad Sci. 2001;936(1):406‐425. 11460495

[34] Jia T , Jacquet T , Dalonneau F , et al. FGF‐2 promotes angiogenesis through a SRSF1/SRSF3/SRPK1‐dependent axis that controls VEGFR1 splicing in endothelial cells. BMC Biol. 2021;19(1):1‐26. 34433435 10.1186/s12915-021-01103-3 PMC8390225

[35] Wang M , Zhang G , Zhang Y , et al. Fibrinogen alpha chain knockout promotes tumor growth and metastasis through integrin–AKT signaling pathway in lung cancer. Mol Cancer Res. 2020;18(7):943‐954. 32205365 10.1158/1541-7786.MCR-19-1033

[36] Zheng S , Shen J , Jiao Y , et al. Platelets and fibrinogen facilitate each other in protecting tumor cells from natural killer cytotoxicity. Cancer Sci. 2009;100(5):859‐865. 19302289 10.1111/j.1349-7006.2009.01115.x PMC11158185

[37] Chen C , Li J , Li J , et al. Application of an elevated plasma D‐dimer cut‐off value improves prognosis prediction of advanced non‐small cell lung cancer. Ann Transl Med. 2020;8(18):1153. 33241002 10.21037/atm-20-5947 PMC7576026

[38] Ma M , Cao R , Wang W , et al. The D‐dimer level predicts the prognosis in patients with lung cancer: a systematic review and meta‐analysis. J Cardiothorac Surg. 2021;16(1):1‐11. 34454552 10.1186/s13019-021-01618-4 PMC8399789

[39] Ma X , Li Y , Zhang J , Huang J , Liu L . Prognostic role of D‐dimer in patients with lung cancer: a meta‐analysis. Tumor Biol. 2014;35(3):2103‐2109. 10.1007/s13277-013-1279-9 24114016

[40] Komurcuoglu B , Ulusoy S , Gayaf M , Guler A , Ozden E . Prognostic value of plasma D‐dimer levels in lungcarcinoma. Tumor J. 2011;97(6):743‐748. 10.1177/030089161109700611 22322841

[41] İNAL S , Taşçi C , Karadurmuş N , et al. The association of D‐dimer levels withother prognostic factors in patients with lung cancer. Turk J Med Sci. 2008;38(3):209‐217.

[42] Yanhua L , Suju W , Junyan W , Lei H , Lige C , Cai W . Analysis of the factors associated with abnormal coagulationand prognosis in patients with non‐small cell lung cancer. Zhongguo Fei Ai Za Zhi. 2014;17(11). 10.3779/j.issn.1009-3419.2014.11.04 PMC6000357 25404269

[43] Qi Y , Fu J . Research on the coagulation function changes in non‐small cell lung cancer patients and analysis oftheir correlation with metastasis and survival. J buon. 2017;22(2):462‐467. 28534370

[44] Van Wersch J , Tjwa M . Coagulation/fibrinolysis balance and lung cancer. Pathophysiology of Haemostasis and Thrombosis. 1991;21(2):117‐123. 10.1159/000216214 1959797

[45] Wang Z‐W , Ye P‐J . Clinical analysis of acute cerebral infarction accompanied with lung cancer. J Acute Dis. 2016;5(4):307‐310.

