# Supplementary figures and images for: Coagulation parameters in lung cancer patients: A systematic review and meta‐analysis
Source: J Clin Lab Anal. 2022 Jun 19;36(7):e24550. doi: 10.1002/jcla.24550 (PMC9279983; doi:10.1002/jcla.24550)

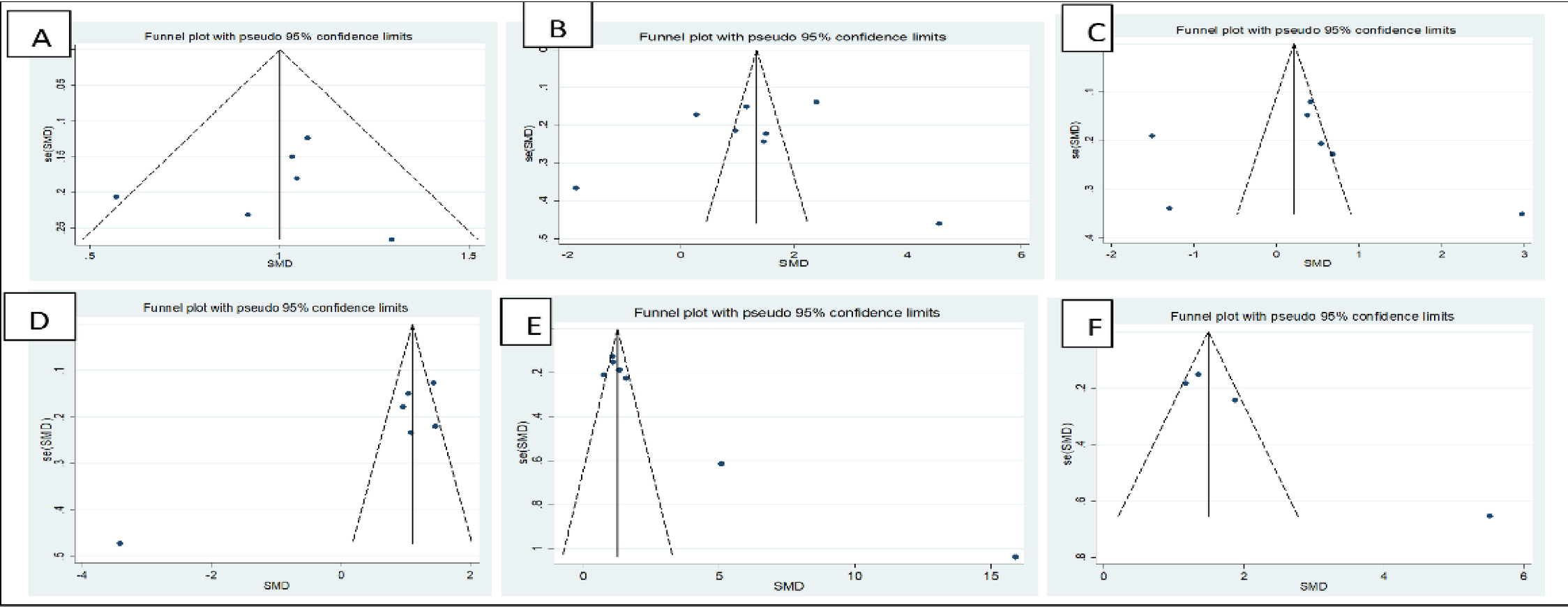

Supplement: Supplementary file 1 — Figure S1 [file JCLA-36-e24550-s006.tif]
