# Supplementary material for: Coagulation parameters in lung cancer patients: A systematic review and meta‐analysis
Source: J Clin Lab Anal. 2022 Jun 19;36(7):e24550. doi: 10.1002/jcla.24550 (PMC9279983; doi:10.1002/jcla.24550)
Supplement: Supplementary file 2 — Table S1 [file JCLA-36-e24550-s002.docx]

Table S1. Egger’s test.

| **Variabls** | **Std_Eff** | **Coef.** | **Std. Err.** | **t** | **P>\|t\|** | **95% CI** |
| --- | --- | --- | --- | --- | --- | --- |
| PLT | Slope | 1.13247 | 0.340751 | 3.32 | 0.029 | 0.1863936-2.078547 |
|  | Bias | -0.7701648 | 1.952366 | -0.39 | 0.713 | -6.190801-4.650471 |
| PT | Slope | 1.760299 | 1.296936 | 1.36 | 0.224 | -1.41319-4.933788 |
|  | Bias | -2.229883 | 6.519228 | -0.34 | 0.744 | -18.18186-13.72209 |
| APTT | Slope | 0.0401033 | 1.216513 | 0.03 | 0.975 | -3.087042-3.167249 |
|  | Bias | 0.9859343 | 6.537982 | 0.15 | 0.886 | -15.82048-17.79235 |
| INR | Slope | 2.782794 | 0.6640836 | 4.19 | 0.014 | 0.9390023-4.626586 |
|  | Bias | -9.824042 | 3.652983 | -2.69 | 0.055 | -19.96635-0.3182648 |
| D-dimer | Slope | -0.7138778 | 0.5329922 | -1.34 | 0.238 | -2.083978-0.6562224 |
|  | Bias | 11.43523 | 2.740007 | 4.17 | 0.009 | 4.39182-18.47864 |
| Fibrinogen | Slope | -0.0675744 | 0.3524227 | -0.19 | 0.866 | -1.583927-1.448778 |
|  | Bias | 8.145923 | 1.695754 | 4.80 | 0.041 | 0.849682-15.44216 |
