# Supplementary material for: Coagulation parameters in lung cancer patients: A systematic review and meta‐analysis
Source: J Clin Lab Anal. 2022 Jun 19;36(7):e24550. doi: 10.1002/jcla.24550 (PMC9279983; doi:10.1002/jcla.24550)
Supplement: Supplementary file 3 — Table S2 [file JCLA-36-e24550-s005.docx]

Table S2: Sensitivity analysis.

| Variable | Excluded study | SMD (95% CI) | Heterogeneity | |
| --- | --- | --- | --- | --- |
|  |  |  | I^2^ | p-value |
| PT | Ujjan et al,2009 | 0.88(0.1-1.66) | 96.6% | 0.000 |
|  | Komurcuoglu et al,2011 | 1.27(0.34-2.20) | 97.4% | 0.000 |
|  | Inal et al,2015 | 1.26(0.33-2.20) | 97.4% | 0.000 |
|  | Yanhua et al,2014 | 1.32(0.31-2.32) | 97.3% | 0.000 |
|  | Yongjun et al,2017 | 1.12(0.29-1.96) | 96% | 0.000 |
|  | Tas et al,2021 | 1.44(0.57-2.32) | 96.7 | 0.000 |
|  | van Wersch et al,1991 | 1.34(0.41-2.28) | 97.3 | 0.000 |
|  | Wang et al,2016 | 1.70(0.97-2.43) | 96 | 0.000 |
| APTT | Ujjan et al,2009 | -0.11(-0.80-0.58) | 95.4 | 0.000 |
|  | Komurcuoglu et al, 2011 | 0.24(-0.66-1.13) | 97% | 0.000 |
|  | Yanhua et al,2014 | 0.28(-0.70-1.28) | 97.1 | 0.000 |
|  | Yongjun et al, 2017 | 0.28(-0.75-1.31) | 97 | 0.000 |
|  | Tas et al,2021 | 0.6(-0.04-1.24) | 93.7 | 0.000 |
|  | van Wersch et al,1991 | 0.26(-0.66-1.77) | 97.1 | 0.000 |
|  | Wang et al,2016 | 0.55(-0.27-1.36) | 96.7 | 0.000 |
| INR | Komurcuoglu et al,2011 | 0.44(-0.39-1.26) | 96 | 0.000 |
|  | Inal et al,2015 | 0.36(-0.46-1.18) | 95.9 | 0.000 |
|  | Yanhua et al,2014 | 0.42(-0.49-1.33) | 96 | 0.000 |
|  | Yongjun et al,2017 | 0.34(-0.57-1.24) | 95.6 | 0.000 |
|  | Tas et al,2021 | 0.45(-0.42-1.31) | 96 | 0.000 |
|  | Wang et al,2016 | 1.20(0.98-1.41) | 59 | 0.00 |
| D-dimer | Komurcuoglu et al,20111 | 1.52(1.02-2.01) | 89.9 | 0.000 |
|  | Inal et al,2015 | 3.47(2.27-4.66) | 98 | 0.000 |
|  | Yanhua et al,2014 | 3.66(2.34-4.97) | 98 | 0.000 |
|  | Yongjun et al,2017 | 3.70(2.33-5.08) | 98 | 0.000 |
|  | Tas et al,2021 | 3.56(2.31-4.80) | 98 | 0.000 |
|  | van Wersch et al,1991 | 3.62(2.42-4.82) | 97.9 | 0.000 |
|  | Wang et al,2016 | 2.72(1.70-3.74) | 97.6 | 0.000 |
| Fibrinogen | Yanhua et al,2014 | 2.56(1.55-3.58) | 95.3 | 0.000 |
|  | Tas et al, 2021 | 2.58(1.64-3.52) | 95.1 | 0.000 |
|  | van Wersch et al,1991 | 2.40(1.42-3.39) | 96.2 | 0.000 |
|  | Wang et al,2016 | 1.73(11.05-2.40) | 93.7 | 0.000 |
|  | Yongjun et al,2017 | 2.18(1.30-3.06) | 93.2 | 0.000 |
