# Supplementary material for: Coagulation parameters in lung cancer patients: A systematic review and meta‐analysis
Source: J Clin Lab Anal. 2022 Jun 19;36(7):e24550. doi: 10.1002/jcla.24550 (PMC9279983; doi:10.1002/jcla.24550)
Supplement: Supplementary file 5 — Appendix S2 [file JCLA-36-e24550-s003.docx]

**Total studies searched form PubMed**

| searches | Query | Result |
| --- | --- | --- |
| #1 | **(((((coagulation parameters[Title/Abstract]) OR (coagulation profile[Title/Abstract])) OR (prothrombin time[Title/Abstract])) OR (activated partial thrombin time[Title/Abstract])) OR (D-dimer[Title/Abstract])) OR (fibrinogen[Title/Abstract])) OR (platelet count[Title/Abstract])** | [95,123](https://pubmed.ncbi.nlm.nih.gov/?term=%28%28%28%28%28%28coagulation+parameters%5BTitle%2FAbstract%5D%29+OR+%28coagulation+profile%5BTitle%2FAbstract%5D%29%29+OR+%28prothrombin++time%5BTitle%2FAbstract%5D%29%29+OR+%28activated++partial+thrombin+time%5BTitle%2FAbstract%5D%29%29+OR+%28D-dimer%5BTitle%2FAbstract%5D%29%29+OR+%28fibrinogen%5BTitle%2FAbstract%5D%29%29+OR+%28platelet+count%5BTitle%2FAbstract%5D%29&sort=) |
| #2 | **((((((coagulation parameters[MeSH Terms]) OR (coagulation profile[MeSH Terms])) OR (prothrombin time[MeSH Terms])) OR (activated partial thrombin time[MeSH Terms])) OR (D-dimer[MeSH Terms])) OR (fibrinogen[MeSH Terms])) OR (Platelet count[MeSH Terms])** | [99,987](https://pubmed.ncbi.nlm.nih.gov/?term=%28%28%28%28%28%28coagulation+parameters%5BMeSH+Terms%5D%29+OR+%28coagulation+profile%5BMeSH+Terms%5D%29%29+OR+%28prothrombin+time%5BMeSH+Terms%5D%29%29+OR+%28activated+partial+thrombin+time%5BMeSH+Terms%5D%29%29+OR+%28D-dimer%5BMeSH+Terms%5D%29%29+OR+%28fibrinogen%5BMeSH+Terms%5D%29%29+OR+%28Platelet+count%5BMeSH+Terms%5D%29&sort=) |
| #3 | #1 OR #2  **(((((((coagulation parameters[Title/Abstract]) OR (coagulation profile[Title/Abstract])) OR (prothrombin time[Title/Abstract])) OR (activated partial thrombin time[Title/Abstract])) OR (D-dimer[Title/Abstract])) OR (fibrinogen[Title/Abstract])) OR (platelet count[Title/Abstract])) OR (((((((coagulation parameters[MeSH Terms]) OR (coagulation profile[MeSH Terms])) OR (prothrombin time[MeSH Terms])) OR (activated partial thrombin time[MeSH Terms])) OR (D-dimer[MeSH Terms])) OR (fibrinogen[MeSH Terms])) OR (Platelet count[MeSH Terms]))** | [149,760](https://pubmed.ncbi.nlm.nih.gov/?term=%28%28%28%28%28%28%28coagulation+parameters%5BTitle%2FAbstract%5D%29+OR+%28coagulation+profile%5BTitle%2FAbstract%5D%29%29+OR+%28prothrombin+time%5BTitle%2FAbstract%5D%29%29+OR+%28activated+partial+thrombin+time%5BTitle%2FAbstract%5D%29%29+OR+%28D-dimer%5BTitle%2FAbstract%5D%29%29+OR+%28fibrinogen%5BTitle%2FAbstract%5D%29%29+OR+%28platelet+count%5BTitle%2FAbstract%5D%29%29+OR+%28%28%28%28%28%28%28coagulation+parameters%5BMeSH+Terms%5D%29+OR+%28coagulation+profile%5BMeSH+Terms%5D%29%29+OR+%28prothrombin+time%5BMeSH+Terms%5D%29%29+OR+%28activated+partial+thrombin+time%5BMeSH+Terms%5D%29%29+OR+%28D-dimer%5BMeSH+Terms%5D%29%29+OR+%28fibrinogen%5BMeSH+Terms%5D%29%29+OR+%28Platelet+count%5BMeSH+Terms%5D%29%29&sort=) |
| #4 | **Lung cancer[Title/Abstract]** | [176,422](https://pubmed.ncbi.nlm.nih.gov/?term=Lung+cancer%5BTitle%2FAbstract%5D&sort=) |
| #5 | **Lung cancer[MeSH Terms]** | [251,632](https://pubmed.ncbi.nlm.nih.gov/?term=Lung+cancer%5BMeSH+Terms%5D&sort=) |
| #6 | #4 OR #5  **(Lung cancer[Title/Abstract]) OR (Lung cancer[MeSH Terms])** | [306,931](https://pubmed.ncbi.nlm.nih.gov/?term=%28Lung+cancer%5BTitle%2FAbstract%5D%29+OR+%28Lung+cancer%5BMeSH+Terms%5D%29&sort=) |
| #7 | #3 AND #6  **((((((((coagulation parameters[Title/Abstract]) OR (coagulation profile[Title/Abstract])) OR (prothrombin time[Title/Abstract])) OR (activated partial thrombin time[Title/Abstract])) OR (D-dimer[Title/Abstract])) OR (fibrinogen[Title/Abstract])) OR (platelet count[Title/Abstract])) OR (((((((coagulation parameters[MeSH Terms]) OR (coagulation profile[MeSH Terms])) OR (prothrombin time[MeSH Terms])) OR (activated partial thrombin time[MeSH Terms])) OR (D-dimer[MeSH Terms])) OR (fibrinogen[MeSH Terms])) OR (Platelet count[MeSH Terms]))) AND ((Lung cancer[Title/Abstract]) OR (Lung cancer[MeSH Terms]))** | [1,153](https://pubmed.ncbi.nlm.nih.gov/?term=%28%28%28%28%28%28%28%28coagulation+parameters%5BTitle%2FAbstract%5D%29+OR+%28coagulation+profile%5BTitle%2FAbstract%5D%29%29+OR+%28prothrombin+time%5BTitle%2FAbstract%5D%29%29+OR+%28activated+partial+thrombin+time%5BTitle%2FAbstract%5D%29%29+OR+%28D-dimer%5BTitle%2FAbstract%5D%29%29+OR+%28fibrinogen%5BTitle%2FAbstract%5D%29%29+OR+%28platelet+count%5BTitle%2FAbstract%5D%29%29+OR+%28%28%28%28%28%28%28coagulation+parameters%5BMeSH+Terms%5D%29+OR+%28coagulation+profile%5BMeSH+Terms%5D%29%29+OR+%28prothrombin+time%5BMeSH+Terms%5D%29%29+OR+%28activated+partial+thrombin+time%5BMeSH+Terms%5D%29%29+OR+%28D-dimer%5BMeSH+Terms%5D%29%29+OR+%28fibrinogen%5BMeSH+Terms%5D%29%29+OR+%28Platelet+count%5BMeSH+Terms%5D%29%29%29+AND+%28%28Lung+cancer%5BTitle%2FAbstract%5D%29+OR+%28Lung+cancer%5BMeSH+Terms%5D%29%29&sort=) |
