# Supplementary material for: Coagulation parameters in lung cancer patients: A systematic review and meta‐analysis
Source: J Clin Lab Anal. 2022 Jun 19;36(7):e24550. doi: 10.1002/jcla.24550 (PMC9279983; doi:10.1002/jcla.24550)
Supplement: Supplementary file 6 — Appendix S3 [file JCLA-36-e24550-s001.docx]

**S1 Quality assessment of enrolled studies for analysis of coagulation abnormalities between lung cancer patients and control group using modified Newcastle - Ottawa quality assessment scale for case- control studies.**

| **Author** | **Criteria** | | | | | | | | |
| --- | --- | --- | --- | --- | --- | --- | --- | --- | --- |
|  | **Selection** | | | | **Comparability** | **Exposure** | | | **Score** |
|  | **Q1** | **Q2** | **Q3** | **Q4** | **Q1** | **Q1** | **Q2** | **Q3** |  |
| Ujjan et al, 2009 | * | * | * | * | * | * | * | * | 8 |
| Komurcuoglu et al, 2011 | ***** | ***** | ***** | ***** | ***** | ***** | ***** | ***** | 8 |
| Inal et al,2015 | …….. | … | … |  |  |  |  |  |  |
| Yanhua et al,2014 | ***** | ***** | ***** | ***** | ***** | **No*** | ***** | ***** | 7 |
| Yongjun et al,2017 | ***** | ***** | ***** | ***** | ***** | ***** | ***** | ***** | 8 |
| Tas et al,2021 | ***** | ***** | ***** | ***** | ***** | ***** | ***** | ***** | 8 |
| van Wersch et al,1991 | ***** | ***** | ***** | ***** | ***** | ***** | ***** | ***** | 8 |
| Wang et al,2016 | ***** | ***** | ***** | ***** | ***** | ***** | ***** | ***** | 8 |

**NB: Score: (*=1 no*=0)**

For those case- control study, we have applied the following questions which have three parts (selection, comparability and exposure) with its decision rules.

| **No.** | **Criterion** | **Decision rule** | **Score: (*=1 no*=0)** |  |
| --- | --- | --- | --- | --- |
| **SELECTION** | | | | |
| 1 | Is the case definition adequate? | 1. Yes, with independent validation (>1 person/record/time/process to extract information, or reference to primary record source such as x-rays or structured injury data)* 2. Yes, based on self-reports 3. No description |  |  |
| 2 | Representativeness of the cases | 1. All eligible cases with outcome of interest over a defined period of time, all cases in a defined catchment area, all cases in a defined team/competition/sport, or a random sample of those cases* 2. Not satisfying requirements in part (a), or not stated. |  |  |
| 3 | Selection of controls | 1. Controls were selected from the same source population as the cases* 2. controls were selected from a different source population 3. no description |  |  |
| 4 | Definition of controls | 1. If cases are first occurrence of injury of interest, then it must explicitly state that controls have no history of this outcome. If cases have new (not necessarily first) occurrence of specific injury, then controls with previous occurrences of outcome of interest should not be excluded* 2. No description of injury history |  |  |
| **COMPARABILITY** | | | | |
| 1 | Comparability of cases and controls on the basis of the design or analysis | 1. Study controls for previous injury* 2. Study controls for age*   *Note:* Cases and controls must be matched in the design and/or confounders must be adjusted for in the analysis. Alone statements of no differences between groups or that differences were not statistically significant are not sufficient. |  |  |
| **EXPOSURE** | | | | |
| 1 | Ascertainment of exposure | 1. Structured injury data (e.g. record completed by medical staff)* 2. Structured interview where blinded to case/control status* 3. Interview not blinded to case/control status 4. Written self-report or medical record (unstructured data) only 5. No description |  |  |
| 2 | Same method of ascertainment for cases and controls | 1. Yes* 2. No |  |  |
| 3 | Non-response rate | 1. Same for both groups* 2. Non-respondents described 3. Rate different and no designation |  |  |
